# Benchmarking of gastric cancer sensitivity to anti-cancer drugs ex vivo as a basis for drug selection in systemic and intraperitoneal therapy

**DOI:** 10.1186/s13046-014-0110-9

**Published:** 2014-12-21

**Authors:** Bo Hultman, Haile Mahteme, Magnus Sundbom, Martin Ljungman, Rolf Larsson, Peter Nygren

**Affiliations:** Department of Surgical Sciences, Uppsala University, Uppsala, Sweden; Surgery Department, Västmanlands Hospital, SE-721 89 Västerås, Sweden; Department of Medical Sciences, Uppsala University, Uppsala, Sweden; Department of Radiology, Oncology and Radiation Sciences, Uppsala University, SE-751 85 Uppsala, Sweden

**Keywords:** Gastric cancer, Anti-cancer drug, Tumor cell, Ex vivo

## Abstract

**Background:**

The choice of drugs for treatment of advanced gastric cancer (GC) is empirical. The purpose of the current study was to benchmark ex vivo the sensitivity of GC tumor cells from patients to standard cytotoxic and some newly introduced targeted drugs (TDs), as a basis for drug selection in the treatment of GC.

**Methods:**

Tumor cell samples from patients with GC were analyzed for sensitivity to 5-fluorouracil, cisplatin, oxaliplatin, irinotecan, mitomycin C, doxorubicin and docetaxel as well as for the targeted drugs bortezomib, sorafenib, sunitinib and rapamycin using a short-term in vitro assay based on retention of viable tumor cells of fluorescent fluorescein. Samples of normal mononuclear cells, chronic lymphocytic leukemia, ovarian cancer and colorectal cancer were included for comparison.

**Results:**

The GC samples were essentially as sensitive to the standard drugs and the TDs as those from colorectal cancer whereas the ovarian cancer samples were more sensitive. The individual GC samples varied considerably in sensitivity to increasing concentrations of the clinically used standard drugs. In GC, cisplatin was cross-resistant to oxaliplatin and 5-fluorouracil which, on the other hand, was not cross-resistant to the other cytotoxic drugs. The activity of sunitinib did not obviously correlate to that of the standard drugs.

**Conclusion:**

Ex vivo assessment of drug sensitivity of tumor cells from patients with GC is feasible and may provide information that could be useful for selection of drugs for treatment. Drug sensitivity varies considerably between and within individual samples arguing for individualized selection of drugs for chemotherapy.

## Background

Gastric cancer (GC) is a tumor type difficult to treat, with high relapse rate following curative surgery [1] and short median survival in the metastatic setting [2]. Empirical testing in the clinic has developed palliative and adjuvant chemotherapy treatment, although there is no fully established standard. A fluoropyrimidine combined with a platinum is mostly used and was previously reported to provide a median overall survival within clinical trials of approximately 11 months [3]. Use of an anthracycline in the 1^st^ line setting provides some minor additional benefit and both docetaxel and irinotecan have been shown to have a role in the 1^st^ or 2^nd^ line treatment settings [4-7].

Among targeted drugs, the HER2 binding monoclonal antibody trastuzumab improves the median overall survival by 2 - 3 months in advanced gastric or gastro-oesophageal junction cancer when added to standard chemotherapy, provided the tumor cells express significant amount of the antibody target [8]. Other targeted drugs (TDs), e g everolimus, sorafenib, sunitinib and bortezomib have been tried in small early clinical trials as single agents or combined with cytotoxic drugs and show varying results in advanced GC, from no to some activity [9-12]. Optimal use of the currently established drugs is now associated with an overall survival (OS) within clinical trials of approximately 14 - 16 months [8,13].

Recent studies also indicate that chemotherapy and/or radiochemotherapy in the perioperative period provides a survival benefit in the curative setting [14,15]. Benefit may also be obtained from intraperitoneal chemotherapy (IPC) added to surgery in this setting [16]. Results from two studies have shown that IPC in combination with systemic chemotherapy, so called bidirectional chemotherapy, may produce long-term survival in peritoneal metastases (PM) from GC [17,18]. Several studies on cytoreductive surgery (removal of macroscopic tumor growth, CRS) in combination with hyperthermic intraperitoneal chemotherapy (HIPEC) indicate benefit from this treatment compared to systemic chemotherapy or CRS without HIPEC [19,20], although not all studies report an obvious benefit [21]. There is little evidence behind the choice of drugs for IPC, which is so far more established in treatment pseudomyxoma peritonei and PM from colorectal cancer (CRC) origin than from GC. Oxaliplatin, doxorubicin, cisplatin, mitomycin C or irinotecan are the drugs mostly used for HIPEC [22,23].

A more differential approach to drug selection for the IPC in PM could provide more efficiency and also for systemic treatment, drug sensitivity testing ex vivo could provide a better basis for drug selection in GC as a group as well as in individual patients compared with the standard empirical approach. In the present study the activities of standard cytotoxic GC active drugs and TDs were investigated ex vivo, using a model known to reflect clinical drug activity. The aims were to describe patterns of drug sensitivity between individual patient samples and between various tumor types to provide additional basis for drug selection in systemic and local treatment of GC.

## Methods

### Patients, sampling and preparation

Tumor samples were collected intraoperatively during primary tumor surgery or by biopsy/surgery if advanced disease from patients with GC, ovarian cancer or CRC. The fraction of samples from patients previously treated with chemotherapy was 75, 15 and 76% in these diagnoses, respectively. In CRC and ovarian cancer prior treatment status has no or very modest effect on cellular sensitivity to standard drugs (unpublished data). Therefore, it was considered reasonable to present data without consideration of prior treatment status. Characteristics of the GC samples (Table 1) were obtained from the patient files. Normal mononuclear cells (MNCs) and chronic lymphocytic leukemia (CLL) cells from chemotherapy naïve patients and known to be generally drug sensitive, were included for reference. The Uppsala regional ethics committee approved the study with the document identifier 2007/237.

**Table 1:** **Characteristics of the gastric cancer patient tumour samples analyzed for drug sensitivity**

**Sex**	**Age at tumour sampling**	Stage at tumour sampling*	**Histology**	**Prior chemotherapy**
Male	39	Metastatic	Signet-ring cell	Yes
Female	58	Metastatic	Signet-ring cell	Yes
Male	60	Metastatic	Intestinal	Yes
Male	58	Localized	Diffuse	Yes
Female	71	Localized	Diffuse	Yes
Female	54	Metastatic	Signet-ring cell	Yes
Male	66	Localized	Intestinal	No
Male	75	Localized	Intestinal	Yes
Female	67	Metastatic	Diffuse	Yes
Male	74	Localized	Intestinal	Yes
Female	70	Localized	Intestinal	No
Male	70	Localized	Intestinal	Yes
Male	70	Localized	Intestinal	Yes
Female	61	Localized	Intestinal	No
Female	88	Localized	Intestinal	No
Male	74	Localized	Intestinal	Yes

*Patients with localized disease underwent curative surgery and tumour sampling was from the primary tumour. Patients with metastatic disease had carcinosis, were planned for cytoreductive surgery and intraperitoneal chemotherapy and tumour sampling was from carcinosis.

Solid tumor tissue was first minced to mm^3^ pieces followed by collagenase digestion [24]. Small cell clusters or single cells from the solid tumors with < 30% contaminating non-malignant cells and with ≥ 90% viability were obtained in most cases of solid tumors, as estimated by morphological examinations of May-Grünwald-Giemsa-stained cytocentrifugate preparations. Prior to seeding onto culture plates, the cells were washed and re-suspended in complete culture medium. MNCs and CLL cells were collected by centrifugation followed by purification on Ficoll-Hypaque (Pharmacia, Uppsala, Sweden) gradients [25]. Overall, 85% of all samples received complied with the criteria for a successful assay (see below) and was included in this study. The GC samples are further detailed on this aspect in the Results section below.

### Drugs and assessment of drug sensitivity ex vivo

The cytotoxic drugs 5-fluorouracil (5-FU; anti-metabolite; Roche), cisplatin (alkylator; Bristol-Myers Squibb), oxaliplatin (alkylator; Sanofi-Synthelabo), irinotecan (topoisomerase I inhibitor; Pfizer), mitomycin C (alkylator; Bristol-Myers Squibb), doxorubicin (topoisomerase II inhibitor; Pfizer) and docetaxel (tubulin stabilizer; Sanofi-Synthelabo) were from commercially available clinical preparations and were dissolved/diluted according to instructions from the manufacturers. The drugs were tested at three 10-fold dilutions from the maximal concentration (μM) of 1000 for 5-FU, 100 for cisplatin, 100 for oxaliplatin, 1000 for irinotecan, 100 for mitomycin C, 10 for doxorubicin and 100 for docetaxel. The targeted drugs bortezomib (proteasome inhibitor), sorafenib (multi-kinase receptor inhibitor), sunitinib (multi-kinase receptor inhibitor) and rapamycin (m-TOR inhibitor) were all from LC laboratories and were dissolved in DMSO and then further diluted in sterile water and were tested at five-fold dilutions from the maximal concentrations of 100 μM, except for bortezomib that had a maximal concentration of 0.4 μM. Experimental plates were prepared in advance with 5 μl drug at the appropriate concentration/well and the plates were then stored at −70°C until use [26].

The fluorometric microculture cytotoxicity assay (FMCA) was used to measure drug sensitivity in the 384-well plate format as described by Lindhagen et al. [26]. Briefly, tumor cells from patient samples (5,000 cells/well for the solid tumor samples and 40,000 cells per well for MNCs and CLL) in 45 μl were seeded in duplicates in drug-prepared or drug free control wells using a pipetting robot. The culture medium was washed away after 72 h incubation and 50 μl/well of a physiological buffer containing 10 μg/ml of the vital dye fluorescein diacetate (FDA) were added to all wells and the fluorescence from each well after 45 min incubation was measured in a Fluoroscan 2 (Labsystems OY, Helsinki, Finland). The fluorescence signal generated by viable cells is proportional to cell number.

### Quantification of results and quality control

The criteria for a successful assay (acceptable quality) were ≥ 70% tumor cells in the cell preparation before incubation and/or on the assay day, a fluorescence signal in control cultures of ≥ five times mean blank values, and a coefficient of variation of cell survival in control cultures of ≤ 30%. The results received by the viability indicator FDA were displayed as survival index (SI): the fluorescence of test cultures expressed in percentage of control cultures, with blank values subtracted. From the SI-results the 50% inhibitory concentrations (IC_50,_ i e the drug concentration producing a SI of 50%) were calculated using a non-linear regression to a standard sigmoidal dose-response model in GraphPad Prism version 5 (GraphPad Software, San Diego, CA, USA).

### Statistics

Data are presented as mean values + standard error of the mean (SE). P value < 0.05 was considered as statistically significant. One-way ANOVA with Dunnett’s post-test was used for statistical inferences between the mean IC_50_-values of samples from GC, ovarian cancer and CRC and Student’s unpaired *t*-test for comparisons between mean IC_50_-values within categories of GC samples. Spearman rank correlation were used for analyze of cross-resistance between selected drugs. The least squares method was used to calculate the regression line slope. The computer software package GraphPad Prism 5.0 was used for statistical evaluation. No P-value corrections were made for multiple-testing.

## Results

### Patient samples

In total 30 tumor samples of GC were obtained: 16 from surgery of primary tumor and 14 from PM. Sixteen of the GC samples (53%) fulfilled the quality criteria and were thus included for data presentation. The reasons for analysis failure were fungus contamination in 8 samples (all from primary tumor) and in 6 samples there were too few cells/too low cell viability to allow for analysis. The number of samples from ovarian cancer was 34, half from metastasis, from CRC 52, all from metastasis, from MNC 44 and from CLL 13. Compared with the overall technical success rate of 85% for the samples included in the study, that of 53% for GC samples was considerably lower. Due to shortage of cells all samples were not tested for all drugs. The number of samples tested for the different drugs are indicated in the figures and figure legends.

Characteristics of the 16 GC samples are shown in Table 1. Eleven of the samples were from the primary tumour in patients undergoing curative surgery and five from carcinosis. Ten samples showed the intestinal histological subtype and the majority of patients had been treated with chemotherapy prior to tumour sampling.

### Ex vivo drug sensitivity

**Figure 1:**
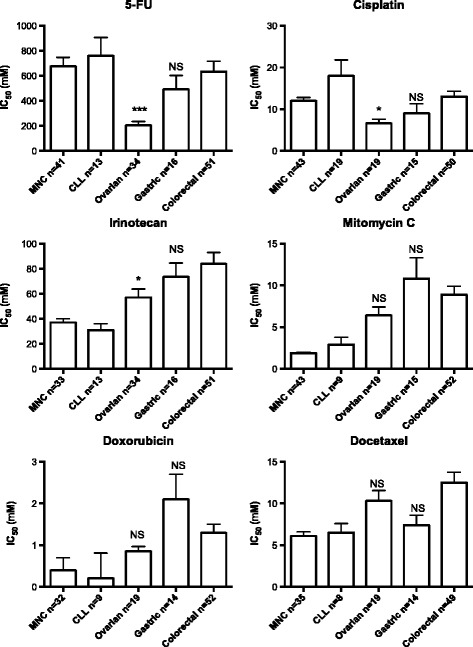
**IC**_**50**_ **values for standard drugs in all tumor samples investigated divided for the subtypes indicated.** Results are presented as mean values + SE. Number of samples investigated for each drug and type of sample is indicated on the x-axis labeling. Statistical interference was calculated with one-way ANOVA with Dunnet’s post-test and with the colorectal cancer samples as reference. Abbeviations: MNC, mononuclear cells; CLL, chronic lymphocytic leukemia; Ovarian, ovarian cancer; Gastric, gastric cancer; Colorectal, colorectal cancer.*P < 0.05; ***P < 0.001; NS, not statistically significant.

Sensitivity to standard drugs, expressed as mean IC_50_ values, of the GC samples did not significantly differ from that of the CRC samples whereas the ovarian cancer samples were more sensitive than the CRC samples to all these drugs, with statistically significant differences for 5-FU, cisplatin and irinotecan (Figure 1). MNC and CLL cells were mostly more sensitive than the solid tumors, except for 5-FU and cisplatin. Notably the GC samples had numerically, although not statistically significantly, lower IC_50_ values than CRC for 5-FU, cisplatin, irinotecan and docetaxel, drugs that are the most clinically active in systemic chemotherapy of GC.

**Figure 2:**
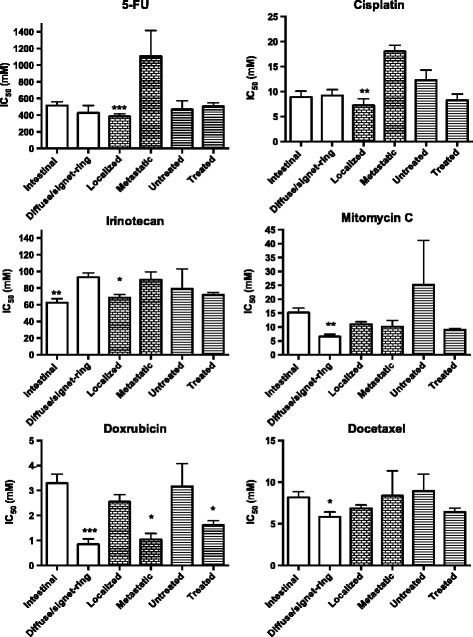
**IC**_**50**_ **values for standard drugs in the gastric cancer samples divided into two groups based on histological subtype, stage at tumour sampling and patient exposure to chemotherapy prior to sampling.** Results are presented as mean values + SE. Number of samples investigated for each drug and sample category was 9 or 10 for intestinal and 5 or 6 for diffuse/signet-ring histology, 11 or 12 for localized and 3 or 4 for metastatic disease, 4 for untreated and 10 – 12 for treated patients. Statistical inference was calculated with Student’s unpaired *t*-test. *P < 0.05; **P < 0.01; ***P < 0.001 with comparison made within each sample category.

Sensitivity to the standard drugs in the GC samples based on histological subtype, stage at tumour sampling and prior chemotherapy is shown in Figure 2. Some statistically significant differences within these categories were observed but the pattern was not consistent. Notably, the notion that the diffuse/signet-ring histological subtype is more drug resistant than the intestinal subtype [27] was not supported by the ex vivo data, rather the contrary. Furthermore, tumour cells from patients previously exposed to chemotherapy were not more resistant than those from chemotherapy naïve patients.

**Figure 3:**
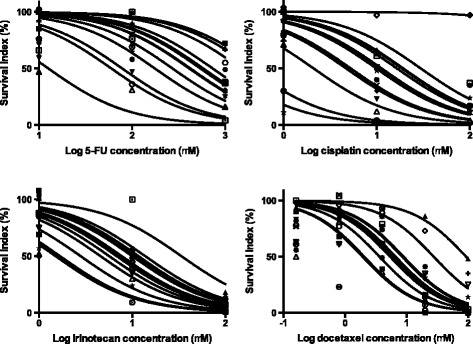
**Display of the concentration - response curves for the individual gastric cancer samples and the indicated drugs.** Considerably inter-individual variability is observed. Curve adaption based on observed data points was made in the GraphPad Prism software. Number of samples investigated was 16 for 5-FU and irinotecan and 15 for cisplatin and docetaxel.

Displaying the individual concentration – response curves for the GC samples for 5-FU, cisplatin, irinotecan and docetaxel revealed considerable inter-individual variability with some samples being sensitive already at the lowest and other samples being resistant also to the highest concentrations tested (Figure 3).

**Figure 4:**
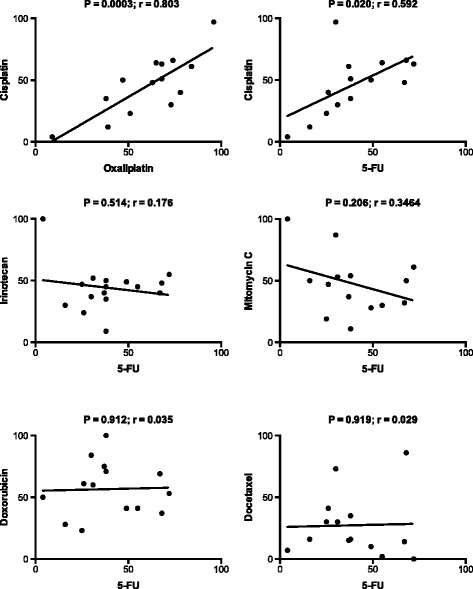
**Correlations between the cytotoxic activites (SI %) for the indicated pairs of standard cytotoxic drugs in gastric cancer samples at concentrations selected to provide optimal activity variation.** These concentrations were: cisplatin, oxaliplatin and mitomycin-C 10 μM; 5-FU and irinotecan 100 μM; doxorubicin 1 μM; docetaxel 20 μM. Abbeviations: r, correlation coefficient; P, the level of statistical significance.

The activity of cisplatin, the platinum drug mostly used in treatment of GC so far, correlated strongly to that of oxaliplatin, in the GC samples (Figure 4). This is in line with recent findings in clinical trials showing oxaliplatin to be at least as active as cisplatin in GC [3], and together these ex vivo and clinical findings provide support for substituting cisplatin with oxaliplatin, a drug more convenient to use, in this tumor type. 5-FU correlated significantly but moderately to cisplatin but not at all to irinotecan, mitomycin C, doxorubicin and docetaxel. These findings indicate that following progression on standard 1^st^ line platinum/5-FU based chemotherapy, at least some activity of these other drugs should be expected in 2^nd^ line treatment, as was also recently shown in clinical trials with irinotecan and docetaxel [5,28].

The observations in Figure 4 that there are many individual samples being resistant to one but sensitive to the other drug support the idea that an optimal choice of drugs for chemotherapy in an individual GC patient should be tailored, e g using ex vivo drug sensitivity testing, rather than standardized as in the currently used strategy.

**Figure 5:**
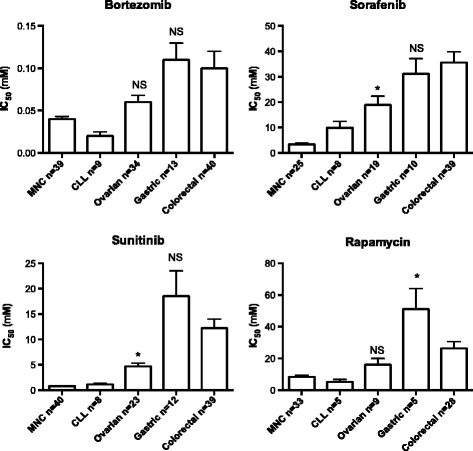
**IC**_**50**_ **values for the targeted drugs bortezomib, sorafenib, sunitinib and rapamycin in all tumor samples investigated divided for the subtypes indicated.** Results are presented as mean values + SE. Number of samples investigated for each drug and type of sample is indicated on the x-axis labeling. Statistical interference was calculated with one-way ANOVA with Dunnet’s post-test and with the colorectal cancer samples as reference. *P < 0.05; NS, not statistically significant.

The pattern of sensitivity to the TDs sorafenib, sunitinib, bortezomib and rapamycin was similar to that of the standard drugs, i e the GC samples showed relatively high IC_50_ values, similar to or even higher than the CRC samples, whereas the ovarian cancer samples, and even more so the MNC and CLL cells were more sensitive (Figure 5). There is yet only very limited experience from these TDs in the clinic in GC although bortezomib was concluded to be inactive whereas sorafenib, sunitinib and mTOR inhibitors similar to rapamycin have been reported to show some activity [9-12]. Our ex vivo data suggests that the activity of these TDs in the clinic is expected to be modest in GC.

**Figure 6:**
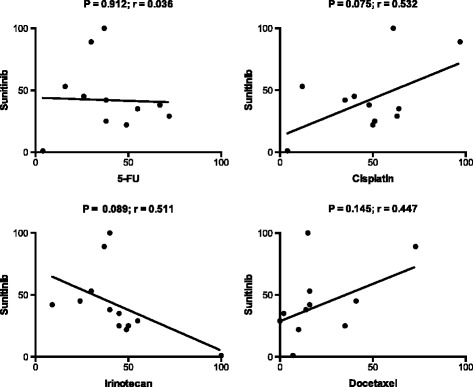
**Correlations between the cytotoxic activites (SI %) for the indicated pairs of standard- and targeted cytotoxic drugs (bortezomib, sorafenib, sunitinib and rapamycin) in gastric cancer samples at concentrations selected to provide optimal activity variation.** These concentrations were: bortezomib 0.08 μM; sorafenib and sunitinib 20 μM; standard drugs as in Figure 4. Abbeviations: r, the correlation coefficient; P, the level of statistical significance.

On the other hand, sunitinib showed low cross-resistance to the GC active standard drugs (Figure 6), indicating that this TD, as well as sorafenib to which sunitinib was significantly cross-resistant (not shown), would be suitable for combination with the standard drugs or for use in 2^nd^ line treatment.

**Figure 7:**
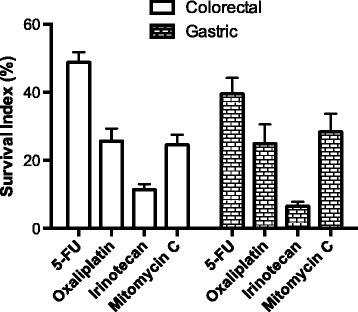
**Tumor cell sensitivity to standard drugs at their maximum concentrations used ex vivo.** These concentrations were: 5-FU and irinotecan 1000 μM; oxaliplatin and mitomycin C 100 μM. Cell survival is expressed as survival index (SI %) in tumor samples from colorectal and gastric cancer. Results are presented as mean values + SE. Number of samples investigated was 15 or 16 for gastric cancer and 51 or 52 for colorectal cancer.

To display what activity against GC tumor cells that could be expected in the IPC situation, in which the tumor cells are exposed to high concentrations of drug, Figure 7 shows the cell survival following exposure to the highest ex vivo concentrations of the drugs commonly used in IPC for CRC. Interestingly the pattern of activity in the GC samples was almost identical to that in the CRC samples. Together with the clinical experience from IPC in CRC, these data provide guidance for selection of drugs for IPC in PM of GC origin.

## Discussion

In solid tumors and in hematological malignancies the FMCA has established ability to predict clinical drug efficacy, both at the diagnosis and the individual patient level [24,25,29]. With this background, we believe that the results from the FMCA analysis in the present study reveal clinically relevant drug sensitivity, although it is admitted that extrapolation from an ex vivo assay to the clinic is difficult considering the added complexity in vivo from factors such as pharmacokinetics and influence on the tumor cells from the surrounding stroma and the immune system. The quite good correspondence between the ex vivo findings presented here for drug sensitivity in GC and the clinical activity for the standard drugs as published provides further support for the clinical relevance of drug sensitivity testing ex vivo using the FMCA.

The present study has the advantage, compared with other published studies on ex vivo testing of GC, of comparing GC with tumor samples from other diagnoses. Such between-diagnosis comparisons are necessary to make reasonable conclusions to the clinic on the activity of drug acitivity to be expected. Tumor samples from GC and CRC in general displayed very similar drug sensitivity, both for the standard drugs and the TDs.

For the standard drugs this corresponds fairly well to the clinical experience that 1^st^ line treatment with similar standard drug combinations in GC and CRC result in similar tumor response rates [30,31], i e tumor cell drug sensitivity per se in these two tumor types is similar, which could be of guidance in the selection of drugs previously not used in one of the tumor types, but from which there is clinical experience in the other. Still, the overall survival of GC and CRC in the advanced settings in recent clinical trials clearly differs, with GC showing median survival in the range 12 – 16 months compared with 20 – 24 months for CRC [8,13,32-34]. Although this might be partly explained by the wider use of 2^nd^ and 3^rd^ line therapy in CRC compared with in GC, the obvious difference also point to tumor biology properties beyond drug sensitivity in gastric cancer [35,36]. Another explanation of the difference in OS for GC and CRC is the possibility of lower stage of disease for CRC compared to GC, at the time of diagnosis.

For the standard drugs the data presented clearly support the substitution in GC of cisplatin for oxaliplatin, which is more convenient to use and which seems at least as active as cisplatin in the clinic [3]. Furthermore, the poor cross-resistance between the standard 1^st^ line agents 5-FU and cisplatin vs irinotecan and docetaxel, provides support for use of the latter drugs in a 2^nd^ line setting. Recent data from clinical trials showing benefit from irinotecan and docetaxel as 2^nd^ line treatment is in agreement with this finding [5,28]. Furthermore, the considerable variability between individual GC samples in sensitivity to increasing drug concentrations and to different drugs clearly gives support for an individual approach for drug selection to optimize drug treatment in GC.

Some efforts in this direction have been tried. Selection of adjuvant chemotherapy for advanced GC was evaluated by Kubota and Weisenthal [37]. Drug sensitive ex vivo corresponded to improved survival. Kim et al. [38] measured ex vivo chemosensitivity in GC using an adenosine triphosphate-based chemotherapy response assay (ATP-CRA). Patients with chemo-naïve advanced GC were treated with a combination of paclitaxel and cisplatin. The ATP-CRA performed well with specificity, sensitivity, positive and negative predictive values being 96, 46, 86, and 76%, respectively. The in vitro chemosensitive group showed higher response rate (86% vs. 24%) compared with the chemoresistant group. However, there were no statistically significant differences in progression free survival or OS. Thus, the ATP-CRA might predict clinical response to paclitaxel and cisplatin with high accuracy. However, these data are retrospective and not validated in an independent cohort, and do not prove that drug selection based on an ex vivo assay would be advantageous compared with the current empirical selection.

A prospective randomized trial comparing therapy based on information from an ex vivo assay compared with the clinicians choice is needed to definitely address this question. The assay should be validated by both accuracy for predicting tumor response and OS, since tumor response is only a surrogate endpoint for OS. Furthermore, not just responders but also patients with stable disease should be included to define chemosensitivity, with an independent review of response evaluation [37].

The outcome for patients with PM of GC origin is dismal and needs to be improved. The pattern of activity in the GC samples in the present study was very similar to that in the CRC samples, supporting the notion of chosing drugs for IPC with proven efficacy for PM of CRC origin also for IPC for PM from GC. Oxaliplatin, cisplatin, mitomycin C, irinotecan and 5-FU are the drugs mostly used for IPC/HIPEC for PM from CRC as well as from GC [19,20,39,40]. Since disease-free survival for IPC/HIPEC is shorter for PM from GC compared to from CRC, it seems reasonable to believe that differences in tumor biology beyond tumor cell drug sensitivity to cytotoxic drugs are behind the dismal OS for PM of GC compared to PM of CRC [19,40].

The current study clearly has some weaknesses. The number of samples was low collected and from a heterogeneous group of patients. The fungus contamination in GC-samples from primary surgery was an obvious methodological problem in present study, limiting the amount of data available. Successful analysis of up to 88% of GC samples has been reported for the histoculture drug response assay [41]. It is essential to improve on the quite low success rate with our technique, to make it more versatile in a clinical setting.

In conclusion, ex vivo assessment of drug activity in GC using the FMCA seems to provide clinically relevant data that could be of guidance in efforts to improve the systemic and intraperitoneal drug treatment of this poor prognosis tumor type. In lack of clinical data on drug activity in GC, the data presented give some support that extrapolation from the experience in CRC is reasonable since these tumor types show similar drug sensitivity profiles ex vivo.
